# Impact of Proton Pump Inhibitor Use on Outcomes Following Carotid Artery Stenting for Asymptomatic Carotid Stenosis: A Population‐Based Cohort Study

**DOI:** 10.1002/kjm2.70178

**Published:** 2026-01-27

**Authors:** Chia‐En Wong, Pang‐Shuo Perng, Pei‐Wen Chen, Yu Chang, Chih‐Hao Tien, Jung‐Shun Lee, Liang‐Chao Wang, Chih‐Yuan Huang

**Affiliations:** ^1^ Division of Neurosurgery, Department of Surgery National Cheng Kung University Hospital, College of Medicine, National Cheng Kung University Tainan Taiwan; ^2^ Center of Transformative Bioelectronic Medicine National Cheng Kung University Tainan Taiwan; ^3^ Department of Cell Biology and Anatomy, College of Medicine National Cheng Kung University Tainan Taiwan; ^4^ Institute of Basic Medical Sciences, College of Medicine National Cheng Kung University Tainan Taiwan

**Keywords:** carotid artery stenting, carotid stenosis, cerebrovascular, proton‐pump inhibitor

## Abstract

Carotid artery stenting (CAS) is an effective treatment for carotid stenosis. Proton‐pump inhibitors (PPIs) are commonly prescribed in the general population. However, the impact of PPI use on outcomes following CAS remains unknown. This study investigated the impact of PPI use on CAS using a retrospective, matched‐cohort analysis from the TriNetX research network. Propensity score matching (PSM) was employed to create two balanced cohorts consisting of regular PPI users and nonusers who underwent CAS for asymptomatic carotid stenosis. Odds ratios (ORs) and 95% confidence intervals (CIs) were calculated using the TriNetX platform to compare cerebrovascular and cardiovascular outcomes. A total of 20,153 patients were included. After PSM, 4691 patients were included in both the PPI and non‐PPI cohorts. The mean age at the time of CAS was 70.4 years in both groups. Compared with non‐PPI users, patients in the PPI cohort had a higher incidence of 30‐day periprocedural stroke (OR: 1.35; 95% CI: 1.08–1.69; *p* = 0.009). Analyses of 2‐year outcomes demonstrated that regular PPI users had a higher incidence of ischemic stroke (OR: 1.16; 95% CI: 1.01–1.32; *p* = 0.034), transient ischemic attack (TIA) (OR: 1.30; 95% CI: 1.14–1.49; *p* < 0.001), and myocardial infarction (OR: 1.19; 95% CI: 1.03–1.38; *p* = 0.018) compared with non‐PPI users. In patients undergoing CAS for asymptomatic carotid stenosis, PPI use was associated with an increased risk of periprocedural stroke, as well as a higher incidence of ischemic stroke, TIA, and myocardial infarction over a 2‐year follow‐up period.

## 1 Introduction

Carotid stenosis is one of the major causes of acute ischemic stroke [1]. Although approximately 60% of patients with carotid stenosis remain asymptomatic, the risk of stroke in patients with high‐grade asymptomatic carotid stenosis remains substantial [1, 2]. Carotid artery stenting (CAS) and carotid endarterectomy (CEA) have been established as effective treatments for both symptomatic and asymptomatic carotid stenosis [3, 4]. Although CAS is widely accepted as a safe approach providing good outcomes, periprocedural complications have been reported [5]. Previous analyses have demonstrated that approximately 4% and 2% of patients experience stroke and myocardial infarction in the periprocedural period, respectively [4, 5].

Proton‐pump inhibitors (PPIs) are commonly prescribed for gastric acid‐related disorders such as peptic ulcer and gastroesophageal reflux disease [6]. Studies have revealed an increasing prevalence of PPI prescriptions, ranging from 14% to 23% in the general population [7]. However, emerging evidence has raised concerns regarding the safety of long‐term PPI use, particularly with respect to cardiovascular and cerebrovascular risks [8]. Prior studies have shown that regular PPI use is associated with an increased risk of stroke, with a higher absolute risk observed in individuals with elevated baseline stroke risk [9]. Despite these findings, whether regular PPI use increases the risk of adverse outcomes in patients with carotid stenosis remains unclear. To the best of our knowledge, the impact of PPI use on outcomes following CAS for asymptomatic carotid stenosis has not been previously documented.

In the present study, we aimed to investigate the impact of PPI use on periprocedural and long‐term cardiovascular and cerebrovascular outcomes following CAS for asymptomatic carotid stenosis. We performed a retrospective, propensity score–matched analysis using a large, global database to evaluate the association between PPI use and postoperative as well as 2‐year outcomes following CAS.

## 2 Material and Methods

This retrospective, population‐based cohort study was conducted using the TriNetX research network, which contains continuously updated, de‐identified health records from healthcare organizations (HCOs) worldwide [10]. Specifically, the TriNetX global collaborative network, comprising 128 HCOs, was queried for this study. The study protocol was approved by the appropriate Institutional Review Board.

**FIGURE 1:**
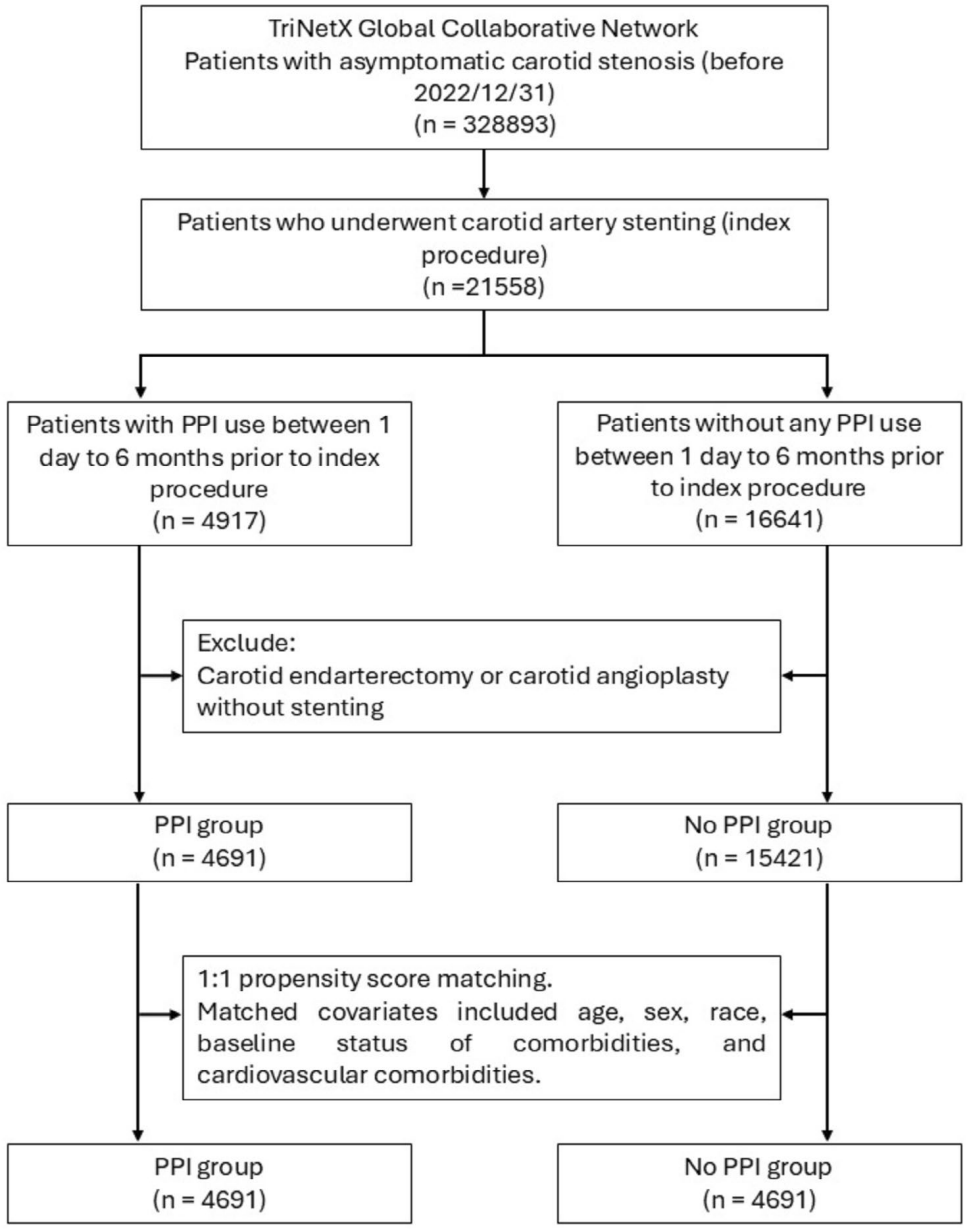
Flowchart of patient selection process from the TriNetX database.

Adult patients diagnosed with carotid artery stenosis and documented in the database from inception through December 2022 were identified. Only individuals aged older than 18 years who underwent CAS were included in the analysis (Figure 1). Individuals who had a diagnosis of ischemic stroke or cerebral infarction before CAS were excluded. Individuals who underwent CEA, open carotid revascularization, or carotid angioplasty without stent placement were also excluded. Given the prospectively updated nature of the database, all included patients had at least 2 years of follow‐up. Detailed diagnostic and procedural codings are provided in Table S1.

Since the present study aimed to investigate the 30‐day periprocedural and 2‐year long‐term outcome after CAS, the index date for outcome analyses was set as the date of the CAS procedure for each patient. Patients with PPI use in the 6 months before the index date were classified into the PPI cohort, while those without PPI use during this period were classified into the non‐PPI control cohort. Patients with a history of previous CAS or CEA procedures before or on the index date were excluded from further analyses.

The primary outcomes were the occurrence of acute ischemic stroke and transient ischemic attack (TIA) during the periprocedural period (30 days) and during long‐term follow‐up (2 years). Secondary outcomes included myocardial infarction, intracerebral hemorrhage, pulmonary embolism, and deep vein thrombosis. Propensity score matching (PSM) was performed to minimize differences in baseline characteristics between the two cohorts. In the primary analysis, odds ratios (ORs) were calculated after full PSM adjustment for age, sex, race, medical comorbidities, and cardiovascular and cerebrovascular comorbidities, including atrial fibrillation and chronic ischemic heart disease (Model 3).

To assess the robustness of the findings, three different PSM models were applied in sensitivity analyses. Model 1 matched patients based on age, sex, and race. Model 2 included age, sex, race, and medical comorbidities such as Type 2 diabetes mellitus, hypertension, and hyperlipidemia. Model 3 incorporated all covariates listed in Table 1.

**TABLE 1:** Propensity matching models.

Models	Model 1	Model 2	Model 3
Matching variables	Age	Age	Age
	Sex	Sex	Sex
	Race	Race	Race
		Type 2 DM	Type 2 DM
		Hypertension	Hypertension
		Hyperlipidemia	Hyperlipidemia
			Atrial fibrillation
			Chronic ischemic heart disease

Statistical analyses were performed using the TriNetX platform's analytic function. In the PSM, baseline characteristics were matched, and the differences of the baseline characteristics were presented using standardized mean differences (SMD), since SMD is essentially independent of sample size, while *p* values are strongly driven by sample size. An SMD value < 0.1 was considered an unremarkable difference between the two groups. ORs for periprocedural and long‐term outcomes were computed to compare the PPI and non‐PPI (control) cohorts. The ORs' statistical significance was determined using 95% confidence intervals (CIs). A *p* < 0.05 was considered statistically significant.

## 3 Results

### 3.1 Baseline Characteristics and PSM

Baseline characteristics of the study cohorts before and after PSM are presented in Table 2. Before matching, 4732 patients were included in the PPI group and 15,421 patients in the non‐PPI group. Significant differences were observed between the groups with respect to ethnicity, Type 2 diabetes mellitus, hypertension, hyperlipidemia, atrial fibrillation, and chronic ischemic heart disease. After PSM, all baseline characteristics demonstrated SMDs of less than 0.1, indicating adequate balance between the matched cohorts.

**TABLE 2:** Baseline characteristics of study subjects before and after propensity score matching.

Characteristics	Before matching		SMD	After matching		SMD
	PPI use *N* = 4732	No PPI *N* = 15,421		PPI use *N* = 4691	No PPI *N* = 4691	
Demographics						
Age	70.4 ± 10.9	70.8 ± 11.0	0.033	70.4 ± 10.9	70.4 ± 10.7	0.003
BMI	28.2 ± 5.9	28.3 ± 5.8	0.013	28.2 ± 5.9	28.6 ± 5.9	0.053
Sex			0.076			0.009
Female	1867	5550		1853	1833	
Male	2712	9438		2711	2732	
Ethnicity			**0.295**			0.083
White	3108	7879		3095	3251	
Black or African American	210	577		209	227	
Asian	86	266		86	85	
Others	1200	6297		1200	1034	
Comorbidities						
Type 2 diabetes	1447	2479	**0.347**	1432	1382	0.023
Hypertension	2883	5619	**0.503**	2868	2860	0.003
Hyperlipidemia	2422	4545	**0.452**	2407	2388	0.008
Atrial fibrillation	755	1133	**0.270**	740	666	0.044
Chronic ischemic heart disease	1972	3678	**0.385**	1957	1927	0.013

*Note:* Bold font represents a standardized difference greater than 0.1.

Abbreviation: SMD: standardized mean difference.

In the matched population, the mean age at the time of CAS was similar between the PPI and non‐PPI groups (70.4 ± 10.9 vs. 70.4 ± 10.7 years, respectively). Female patients comprised 39.5% of the PPI group and 39.1% of the non‐PPI group. Atrial fibrillation was present in 15.8% of patients in the PPI group and 14.2% in the non‐PPI group. Chronic ischemic heart disease was observed in 41.7% and 41.1% of patients in the PPI and non‐PPI groups, respectively.

### 3.2 Periprocedural Outcomes

Outcomes in the 30‐day periprocedural period are shown in Table 3. Patients in the PPI group had a higher incidence of postoperative ischemic stroke compared to patients in the non‐PPI group (PPI group: 3.9% vs. non‐PPI group: 2.9%; OR: 1.35; 95% CI: 1.08–1.69; *p* = 0.009). In addition, there was a nonsignificant trend of higher incidence of myocardial infarction in the PPI group compared to patients in the non‐PPI group (PPI group: 2.0% vs. non‐PPI group: 1.5%; OR: 1.30; 95% CI: 0.95–1.78; *p* = 0.096).

**TABLE 3:** Odds ratio of periprocedural outcomes with 95% confidence interval.

Outcomes	PPI use *N* = 4691		No PPI *N* = 4691		Odds ratio	95% CI	*p*
	*n*	%	*n*	%			
Primary outcomes							
Ischemic stroke	184	3.9	138	2.9	1.35	1.08–1.69	**0.009**
TIA	170	3.6	164	3.5	1.04	0.83–1.29	0.738
Secondary outcomes							
Myocardial infarction	92	2.0	71	1.5	1.30	0.95–1.78	0.097
Intracerebral hemorrhage	18	0.4	16	0.3	1.13	0.57–2.21	0.731
Pulmonary embolism	16	0.3	16	0.3	1.00	0.50–2.00	0.999
Deep vein thrombosis	16	0.3	11	0.2	1.46	0.68–3.14	0.335

*Note:* Bold font represents a *p* < 0.05. OR, 95% CI, and *p* values were estimated from logistic regression after PSM Model 3.

The difference in the percentage of TIA (PPI group: 3.6% vs. non‐PPI group: 3.5%; OR: 1.04; 95% CI: 0.83–1.29; *p* = 0.738), intracerebral hemorrhage (PPI group: 0.4% vs. non‐PPI group: 0.3%; OR: 1.13; 95% CI: 0.57–2.21; *p* = 0.731), pulmonary embolism (PPI group: 0.3% vs. non‐PPI group: 0.3%; OR: 1.00; 95% CI: 0.50–2.00; *p* = 0.999), and deep vein thrombosis (PPI group: 0.3% vs. non‐PPI group: 0.2%; OR: 1.46; 95% CI: 0.68–3.14; *p* = 0.335) were not significant between the two groups.

### 3.3 Long‐Term Outcomes

Long‐term outcomes in the 2‐year follow‐up period are shown in Table 4. Patients in the PPI group had a higher incidence of ischemic stroke (PPI group: 10.7% vs. non‐PPI group: 9.4%; OR: 1.16; 95% CI: 1.01–1.32; *p* = 0.034) and TIA (PPI group: 10.7% vs. non‐PPI group: 8.4%; OR: 1.30; 95% CI: 1.14–1.49; *p* < 0.001) compared to patients in the non‐PPI group. In the secondary outcomes, patients in the PPI group had a higher incidence of myocardial infarction compared to patients in the non‐PPI group (PPI group: 9.1% vs. non‐PPI group: 7.8%; OR: 1.19; 95% CI: 1.03–1.38; *p* = 0.018).

**TABLE 4:** Odds ratio of long‐term outcomes with 95% confidence interval.

Outcomes	PPI use *N* = 4691		No PPI *N* = 4691		Odds ratio	95% CI	*p*
	*n*	%	*n*	%			
Primary outcomes							
Ischemic stroke	504	10.7	442	9.4	1.16	1.01–1.32	**0.034**
TIA	500	10.7	393	8.4	1.30	1.14–1.49	**< 0.001**
Secondary outcomes							
Myocardial infarction	429	9.1	365	7.8	1.19	1.03–1.38	**0.018**
Intracerebral hemorrhage	63	1.3	52	1.1	1.21	0.84–1.76	0.302
Pulmonary embolism	62	1.3	59	1.3	1.05	0.74–1.51	0.784
Deep vein thrombosis	63	1.3	56	1.2	1.13	0.78–1.62	0.519

*Note:* Bold font represents a *p* < 0.05. OR, 95% CI, and *p* values were estimated from logistic regression after PSM Model 3.

The difference in the percentage of intracerebral hemorrhage (PPI group: 1.3% vs. non‐PPI group: 1.1%; OR: 1.21; 95% CI: 0.84–1.76; *p* = 0.302), pulmonary embolism (PPI group: 1.3% vs. non‐PPI group: 1.3%; OR: 1.05; 95% CI: 0.74–1.50; *p* = 0.784), and deep vein thrombosis (PPI group: 1.3% vs. non‐PPI group: 1.2%; OR: 1.13; 95% CI: 0.78–1.62; *p* = 0.519) were not significant between the two groups.

### 3.4 Sensitivity Analysis

**FIGURE 2:**
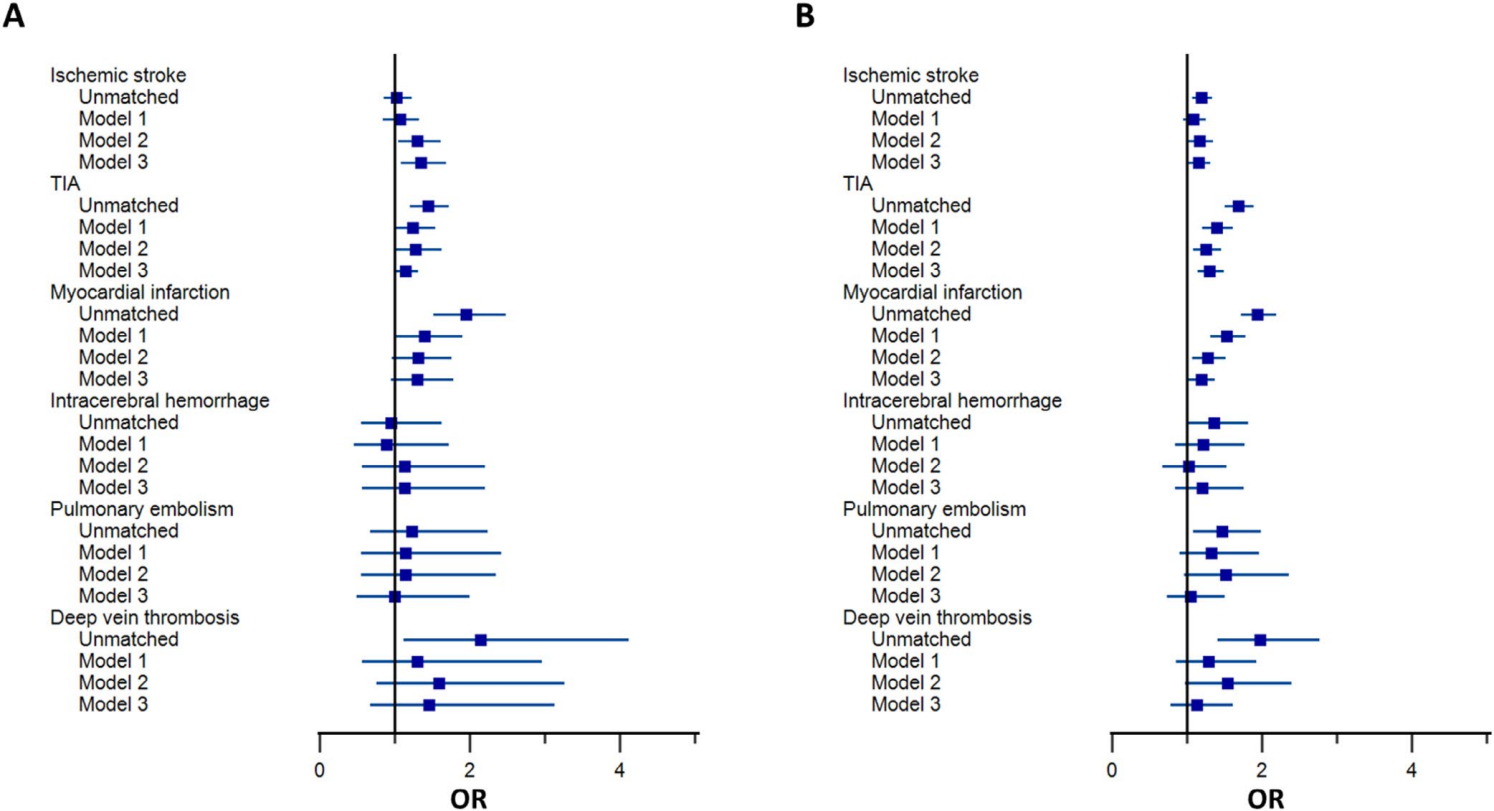
Sensitivity analyses. Sensitivity analyses for (A) 30‐day periprocedural and (B) 2‐year long‐term outcomes.

Sensitivity analyses were performed to evaluate how different PSM strategies influenced the outcomes. In the perioperative outcomes, the analysis of ischemic stroke showed a gradual increase in statistical difference toward higher risks of ischemic stroke after PSM (Figure 2A). The risks of ischemic stroke were significantly higher in Model 2 (OR: 1.30; 95% CI: 1.05–1.62; *p* = 0.017) and Model 3 (OR: 1.35; 95% CI: 1.08–1.69; *p* = 0.009) (Table 5). In the results of TIA, patients with PPI use had a higher risk of TIA in the unmatched analysis (OR: 1.45; 95% CI: 1.21–1.73; *p* < 0.001); however, this association was attenuated after adjusting for PSM in Models 1–3. Likewise, patients with PPI use had a higher risk of myocardial infarction in the unmatched analysis (OR: 1.95; 95% CI: 1.52–2.49; *p* < 0.001) and Model 1 (OR: 1.40; 95% CI: 1.03–1.91; *p* = 0.030); however, this association was attenuated after adjusting for PSM in Models 2 and 3. The sensitivity analyses of intracerebral hemorrhage, pulmonary embolism, and deep vein thrombosis showed similar results in all models.

**TABLE 5:** Sensitivity analysis of periprocedural outcomes with 95% confidence interval.

Outcomes	Unmatched			Model 1			Model 2			Model 3		
	OR	95% CI	*p*	OR	95% CI	*p*	OR	95% CI	*p*	OR	95% CI	*p*
Primary outcomes												
Ischemic stroke	1.03	0.86–1.23	0.719	1.07	0.85–1.33	0.573	1.30	1.05–1.62	**0.017**	1.35	1.08–1.69	**0.009**
TIA	1.45	1.21–1.73	**< 0.001**	1.24	0.99–1.55	0.065	1.28	0.99–1.63	0.057	1.14	0.99–1.32	0.053
Secondary outcomes												
Myocardial infarction	1.95	1.52–2.49	**< 0.001**	1.40	1.03–1.91	**0.030**	1.31	0.97–1.76	0.081	1.30	0.95–1.78	0.097
Intracerebral hemorrhage	0.95	0.55–1.63	0.845	0.89	0.46–1.72	0.738	1.13	0.57–2.21	0.731	1.13	0.57–2.21	0.731
Pulmonary embolism	1.23	0.68–2.24	0.487	1.15	0.55–2.43	0.705	1.14	0.56–2.35	0.715	1.00	0.50–2.00	0.999
Deep vein thrombosis	2.15	1.12–4.12	**0.018**	1.30	0.57–2.97	0.531	1.59	0.76–3.27	0.208	1.46	0.68–3.14	0.335

*Note:* OR, 95% CI, and *p* values were estimated from logistic regressions. Bold values indicate statistically significant *p* < 0.05.

In the sensitivity analyses of long‐term outcomes, the analyses of ischemic stroke, TIA, and myocardial infarction in patients with PPI use compared to non‐PPI users revealed a consistently higher OR across all models, including the unmatched analysis (Figure 2B). Although patients with PPI use had a higher risk of intracerebral hemorrhage (OR: 1.36; 95% CI: 1.01–1.82; *p* = 0.045), pulmonary embolism (OR: 1.47; 95% CI: 1.08–1.99; *p* = 0.013), and deep vein thrombosis (OR: 1.98; 95% CI: 1.41–2.78; *p* = 0.001) compared to non‐PPI users in the unmatched analysis; however, these associations were attenuated after adjusting for PSM in Models 1–3 (Table 6).

**TABLE 6:** Sensitivity analysis of long‐term outcomes with 95% confidence intervals.

Outcomes	Unmatched			Model 1			Model 2			Model 3		
	OR	95% CI	*p*	OR	95% CI	*p*	OR	95% CI	*p*	OR	95% CI	*p*
Primary outcomes												
Ischemic stroke	1.19	1.07–1.34	0.002	1.09	0.95–1.25	0.207	1.17	1.01–1.35	0.039	1.16	1.01–1.32	0.034
TIA	1.69	1.51–1.89	**< 0.001**	1.40	1.21–1.62	**< 0.001**	1.26	1.08–1.46	**0.003**	1.30	1.14–1.49	**< 0.001**
Secondary outcomes												
Myocardial infarction	1.94	1.72–2.20	**< 0.001**	1.53	1.31–1.78	**< 0.001**	1.28	1.07–1.52	**0.007**	1.19	1.03–1.38	**0.018**
Intracerebral hemorrhage	1.36	1.01–1.82	**0.045**	1.22	0.84–1.77	0.298	1.02	0.68–1.53	0.918	1.21	0.84–1.76	0.302
Pulmonary embolism	1.47	1.08–1.99	**0.013**	1.33	0.91–1.97	0.141	1.52	0.97–2.36	0.061	1.05	0.74–1.51	0.784
Deep vein thrombosis	1.98	1.41–2.78	**< 0.001**	1.29	0.86–1.93	0.218	1.54	0.98–2.40	0.058	1.13	0.78–1.62	0.519

*Note:* OR, 95% CI, and *p* values were estimated from logistic regressions. Bold values indicate statistically significant *p* < 0.05.

### 3.5 Subgroup Analyses

Among patients with preoperative PPI use, pantoprazole, esomeprazole, and omeprazole were the most frequently prescribed PPIs, used by 1737, 1472, and 925 patients, respectively. Subgroup analyses were performed to evaluate whether different PPIs were associated with differential outcomes.

In the comparison between pantoprazole and esomeprazole, a nonsignificant trend toward a higher risk of periprocedural stroke was observed in patients using esomeprazole (OR: 0.68; 95% CI: 0.44–1.06; *p* = 0.089). The incidence of periprocedural TIA, myocardial infarction, long‐term ischemic stroke, TIA, myocardial infarction, intracerebral hemorrhage, and deep vein thrombosis was similar between the subgroups (Table S2).

In the analysis of Pantoprazole versus Omeprazole, the differences in periprocedural ischemic stroke, TIA, myocardial infarction, long‐term ischemic stroke, TIA, myocardial infarction, intracerebral hemorrhage, and deep vein thrombosis were not significant between the two subgroups (Table S3).

In addition, we investigated whether a previous myocardial infarction would affect the outcomes following CAS. In the subgroup analysis, previous myocardial infarction was associated with increased 2‐year risk of ischemic stroke (OR: 1.37; 95% CI: 1.10–1.69; *p* = 0.004). The incidence of periprocedural ischemic stroke, TIA, long‐term TIA, intracerebral hemorrhage, and deep vein thrombosis was similar between the subgroups (Table S4).

## 4 Discussion

CAS is an effective treatment for preventing ischemic stroke in patients with carotid stenosis. PPIs are widely prescribed in the general population. In this study, we evaluated the impact of PPI use on 30‐day periprocedural and 2‐year long‐term cerebrovascular and cardiovascular outcomes following CAS for asymptomatic carotid stenosis. Using data from the TriNetX network and a propensity score‐matched design, we found that PPI use was associated with a higher risk of periprocedural ischemic stroke. Furthermore, long‐term analyses demonstrated that PPI users had higher risks of ischemic stroke, TIA, and myocardial infarction compared with non‐PPI users.

Carotid stenosis with > 50% luminal narrowing accounts for approximately 10%–20% of ischemic strokes, with 5‐year cumulative stroke risks ranging from 2% to 25% [11, 12, 13, 14]. These risks were significantly reduced following carotid revascularization with either CAS or CEA, which have demonstrated comparable long‐term efficacy [4]. Current clinical guidelines recommend carotid revascularization for symptomatic patients with greater than 50% stenosis and for asymptomatic patients with greater than 60% stenosis [15, 16]. Furthermore, CAS is increasingly performed as a minimally invasive procedure that does not require general anesthesia, which offers an advantage in individuals with medical comorbidities precluding CEA under general anesthesia [17].

PPI is one of the most commonly prescribed medications worldwide and is available both over the counter and by prescription [7]. Common indications include peptic ulcer, gastroesophageal reflux disease, and other acid‐related disorders [6, 7]. Although PPIs are generally regarded as safe and well‐tolerated, growing evidence has raised concerns regarding their potential adverse cardiovascular and cerebrovascular effects [8, 9, 18]. In a large prospective study involving 492,479 participants, Yang et al. reported that regular PPI use was associated with a 16% increased risk of stroke, with greater absolute risk among individuals with higher baseline stroke risk [9]. Similarly, a meta‐analysis of 41 studies reported a modest increase in stroke risk among individuals without prior cardiovascular disease [18]. Additional studies by Bell et al. and Li et al. have also identified associations between PPI use and an increased incidence of cardiovascular diseases, including coronary heart disease and heart failure [19].

These associations were particularly worrisome in patients with a high baseline risk of cerebrovascular events, such as individuals with carotid stenosis, which is one of the major causes of acute ischemic stroke [20]. Therefore, in this study, we investigated the impact of PPI use on patients undergoing CAS for asymptomatic carotid stenosis. In the Carotid Revascularization Endarterectomy versus Stenting Trial (CREST) clinical trial, 4.1% and 1.1% of patients had stroke and myocardial infarction in the 30‐day periprocedural period following CAS [4]. Herein, we reported similar incidence rates, with 2.9% and 1.5% incidence of periprocedural stroke and myocardial infarction, respectively. Notably, we found that regular PPI use was associated with a higher risk of ischemic stroke following CAS in the 30‐day periprocedural period. Periprocedural stroke is a significant complication following CAS and can be several factors, including plaque dislodgement with distal embolism, intraprocedural hemodynamic disturbance, and stent thrombosis [21, 22]. Studies have shown long‐term PPI use may disrupt endothelial function and nitric oxide pathways, thereby potentially contributing to stent thrombosis and cerebral hypoperfusion [23]. In addition, previous studies have shown that PPI and clopidogrel interact through cytochrome P450 enzyme inhibition, reducing the antiplatelet efficacy [24]. This could potentially contribute to the increased incidence of stroke in PPI users since most patients require dual antiplatelet therapy after CAS, and inadequate antiplatelet therapy significantly increases the risk of ischemic stroke, stent thrombosis, and poor clinical outcomes [25].

In analyses of the 2‐year long‐term outcomes, PPI use was also associated with increased risks of TIA and myocardial infarction in addition to ischemic stroke. PPI‐related endothelial dysfunction may contribute to in‐stent restenosis, thereby increasing the risk of TIA [23]. Furthermore, reduced antiplatelet efficacy due to PPI–clopidogrel interactions may negatively affect long‐term outcomes following CAS, including increased 12‐month stroke risk [26]. In‐stent restenosis, which has a reported 5‐year incidence of approximately 10%–40% depending on severity, is also significantly associated with TIA risk [27, 28].

Carotid artery stenosis is a marker of systemic atherosclerosis, and patients frequently have coexisting cardiovascular disease, including coronary artery disease, which increases the risk of myocardial infarction [29]. The association between regular PPI use and cardiovascular events, including myocardial infarction, is well reported, and it is reasonable that regular PPI users with a higher baseline risk of cardiovascular events might have higher risks of myocardial infarction [23]. Collectively, these findings underscore the increased cerebrovascular and cardiovascular risks observed in PPI users after CAS and support the recommendation for enhanced clinical surveillance in this patient population.

This study had limitations. First, information bias might exist in retrospective database‐based real‐world studies, including missing data, misclassification bias, and potential residual confounding factors [30]. Nonetheless, our sensitivity analysis demonstrated consistent outcomes across different matching models, indicating stable results. Also, because administrative codes were utilized to define diseases, medication prescriptions, and procedures, critical variables such as the degree of carotid stenosis and preprocedural imaging data were not available from the database, which is an inherent limitation of electronic health record database studies. Second, we included only asymptomatic patients with carotid stenosis in this study. Patients with symptomatic carotid stenosis would have the diagnostic codes for stroke or TIA before the index procedure, which could confound outcome analysis since the detection of outcome events was based on the diagnostic coding in the TriNetX database. Furthermore, in the present study, we were unable to investigate the potential impact of PPI on in‐stent restenosis following CAS, which is a significant factor associated with stroke and TIS. Future prospective studies are warranted to evaluate the effect of PPI use on the radiographic outcomes following CAS. Nonetheless, our study has several strengths, including a large sample size, which increased the statistical power and precision of our estimates. Moreover, we utilized PSM to balance baseline characteristics and minimize potential confounding bias.

## 5 Conclusions

In this large, propensity score‐matched, real‐world, database‐driven study, we evaluated the impact of regular PPI use on outcomes following CAS for asymptomatic carotid stenosis. PPI use was associated with a higher risk of ischemic stroke during the 30‐day periprocedural period following CAS. In addition, long‐term analyses demonstrated that PPI users had increased risks of ischemic stroke, TIA, and myocardial infarction compared with non‐PPI users.

## Ethics Statement

This research was approved by the Institutional Review Board of National Cheng Kung University Hospital (NCKU‐IRB‐Approval No. A‐ER‐113‐417).

## Conflicts of Interest

The authors declare no conflicts of interest.

## Supporting information


**Data S1:** Supporting Information.

## Data Availability

The data that support the findings of this study are available on request from the corresponding author. The data are not publicly available due to privacy or ethical restrictions.
