# Supplementary material for: Impact of Proton Pump Inhibitor Use on Outcomes Following Carotid Artery Stenting for Asymptomatic Carotid Stenosis: A Population‐Based Cohort Study
Source: Kaohsiung J Med Sci. 2026 Jan 27;42(8):e70178. doi: 10.1002/kjm2.70178 (PMC13399625; doi:10.1002/kjm2.70178)
Supplement: Supplementary file 1 [file KJM2-42-e70178-s001.pdf]

Supplementary Table 1. Query codes for diagnoses and procedures

Diagnosis	System	Code
Carotid stenosis	ICD-10	I65.2
Carotid artery stenting	ICD-10-PCS	037L3DZ<br>037K3DZ<br>037L34Z<br>037K34Z
Proton pump inhibitors	ATC	A02BC
Omeprazole	RxNorm	7646
Esomeprazole	RxNorm	283742
Pantoprazole	RxNorm	40790
DM	ICD-10	E11
Hypertension	ICD-10	I10-I1A
Hyperlipidemia	ICD-10	E78
Atrial fibrillation	ICD-10	I48
Chronic ischemic heart disease	ICD-10	I25
Ischemic stroke	ICD-10	I63
TIA	ICD-10	G45
Myocardial infarction	ICD-10	I21
Intracerebral hemorrhage	ICD-10	I61
Pulmonary embolism	ICD-10	I26
Deep vein thrombosis	ICD-10	I82<br>I82.4

Supplementary Table 2 Subgroup analysis on Pantoprazole versus Esomeprazole

Outcomes	Pantoprazole N = 1316		Esomeprazole N = 1316		Odds ratio	95% CI	p-value
	n	%	n	%			
**Periprocedural outcomes**							
**Primary outcomes**							
Ischemic stroke	74	5.7	106	8.1	0.68	0.44—1.06	0.089
TIA	50	3.8	58	3.3	0.86	0.50—1.48	0.578
**Secondary outcomes**							
Myocardial infarction	34	2.6	49	3.7	0.69	0.33—1.42	0.161
**Long term outcomes**							
**Primary outcomes**							
Ischemic stroke	146	11.1	162	12.3	0.89	0.64—1.25	0.493
TIA	152	11.6	154	11.7	0.99	0.70—1.38	0.932
**Secondary outcomes**							
Myocardial infarction	102	7.8	135	10.3	0.73	0.50—1.08	0.106
Intracerebral hemorrhage	22	1.7	24	1.8	1.09	0.84—1.56	0.234
Deep vein thrombosis	22	1.7	20	1.5	1.10	0.47—2.61	0.826

Supplementary Table 3 Subgroup analysis on Pantoprazole versus Omeprazole

Outcomes	Pantoprazole N = 729		Omeprazole N = 729		Odds ratio	95% CI	p-value
	n	%	n	%			
**Periprocedural outcomes**							
**Primary outcomes**							
Ischemic stroke	57	7.8	51	7.0	1.13	0.90—1.41	0.300
TIA	38	5.3	41	5.7	0.92	0.71—1.20	0.549
**Secondary outcomes**							
Myocardial infarction	20	2.7	18	2.5	1.11	0.77—1.62	0.569
**Long term outcomes**							
**Primary outcomes**							
Ischemic stroke	82	11.3	84	11.5	0.97	0.80—1.17	0.739
TIA	97	13.4	98	13.5	0.99	0.83—1.18	0.929
**Secondary outcomes**							
Myocardial infarction	64	8.7	67	9.2	0.95	0.77—1.16	0.597
Intracerebral hemorrhage	17	2.3	21	2.8	0.79	0.54—1.15	0.215
Deep vein thrombosis	13	1.8	14	1.9	0.95	0.61—1.48	0.821

Supplementary Table 4 Subgroup analysis on previous myocardial infarction (MI)

Outcomes	Previous MI N = 1891		No MI N = 1891		Odds ratio	95% CI	p-value
	n	%	N	%			
**Periprocedural outcomes**							
**Primary outcomes**							
Ischemic stroke	94	5.1	115	6.3	0.82	0.61—1.09	0.135
TIA	80	4.4	84	4.6	0.95	0.70—1.30	0.749
**Long term outcomes**							
**Primary outcomes**							
Ischemic stroke	217	11.5	164	8.7	1.37	1.10—1.69	**0.004**
TIA	188	9.9	175	9.3	1.08	0.87—1.34	0.473
**Secondary outcomes**							
Intracerebral hemorrhage	43	2.3	36	1.9	1.19	0.77—1.87	0.426
Deep vein thrombosis	39	2.1	38	2.0	1.03	0.65—1.61	0.908
